# London Lancet

**Published:** 1862-07

**Authors:** 


LONDON LANCET. This old and ever welcome foreign monthly for July, is on our table promptly, as usual; and well filled with interesting matter.

